# Contextual and individual determinants of tooth loss in adults: a multilevel study

**DOI:** 10.1186/s12903-020-1057-1

**Published:** 2020-03-17

**Authors:** Luana Leal Roberto, Marise Fagundes Silveira, Alfredo Mauricio Batista de Paula, Efigênia Ferreira e Ferreira, Andréa Maria Eleutério de Barros Lima Martins, Desirée Sant’ana Haikal

**Affiliations:** 1 Postgraduate Program in Health Sciences, State University of Montes Claros (Universidade Estadual de Montes Claros), Montes Claros, Minas Gerais Brazil; 2 Department of Mathematics, State University of Montes Claros (Universidade Estadual de Montes Claros), Montes Claros, Minas Gerais Brazil; 3 Department of Dentistry, State University of Montes Claros (Universidade Estadual de Montes Claros), Montes Claros, Minas Gerais Brazil; 4 Department of Community and Preventive Dentistry, School of Dentistry, Federal University of Minas Gerais (Universidade Federal de Minas Gerais), Belo Horizonte, Minas Gerais Brazil

**Keywords:** Adult, Tooth loss, Health inequalities, Multilevel analysis

## Abstract

**Background:**

Tooth loss represents a known marker of health inequality. The association between tooth loss and unfavorable socioeconomic conditions is evident when analyzed at an individual level. However, the effects of contextual characteristics on tooth loss need to be better investigated and understood. The objective of this study was to analyze tooth loss among Brazilian adults (35–44 years of age), in accordance with individual and contextual social characteristics.

**Methods:**

This was a multilevel cross-sectional study with data from 9564 adult participants from the Brazilian Oral Health Survey - SBBrasil 2010. The dependent variable was the number of lost teeth and the independent variables were grouped into structural (socioeconomic & political context) and intermediary (socioeconomic position, behavioral & biological factors, and health services) determinants. Multilevel Hierarchical Negative Binomial Regression was conducted and the Mean Ratio (MR) was estimated.

**Results:**

Brazilian adults lost a mean of 7.57 (95% CI 7.1–8.1) teeth. Among the contextual variables, the number of teeth lost was higher among residents of municipalities with high and medium/low Municipal Human Development Index (MHDI) and in municipalities that did not have public water fluoridation. Among the individual variables, dental loss was higher among those who declared themselves yellow/black/brown/indigenous, were older, who had lower income, who had never visited a dentist, who had used dental services for more than a year and those whose most recent visit to the dentist was due to oral health problems. On the other hand, dental loss was lower among adults with higher education levels and males.

**Conclusions:**

The number of missing teeth was associated with unfavorable contextual and individual conditions, which reinforces the need to reduce social inequality and guarantee regular, lifetime access to dental services.

## Background

Tooth loss is considered as a major oral health issue [1, 2] and an important public health problem [3]. Besides reflecting the accumulation of oral disease throughout life [4], tooth loss can also be influenced by social, behavioral and cultural factors [3, 5]. Thus, tooth loss is the result of a complex interaction between biological and social factors [6].

Tooth loss is a marker of health disparity in the population [7], based on the fact that socially disadvantaged groups show lower number of the teeth [8]. This association is evident at the individual level [2, 7, 9–11]. However, the effects of the place where people live (contextual effects) on tooth loss needs further investigation to be understood.

The need to recognize and distinguish contextual influences on oral health has been identified in the scientific literature. A systematic review conducted to identify the contextual socioeconomic factors associated with dental loss found that there are very few studies evaluating tooth loss in the contextual perspective [12]. Some studies have reported the contextual characteristics associated with tooth loss, such as Gini coefficient [7, 13], municipal human development index (MHDI) [14], fluoridated water supply [14–16], and place of residence (urban versus rural) [15]. Such studies are essential, since not all determinants of the health-disease process can be captured at the individual level [17].

This study aimed to analyze tooth loss among Brazilian adults, and asses its association with both contextual social inequalities and individual characteristics using a multilevel approach. We hypothesized that the total number of missing teeth in adults is affected by contextual social inequalities even after controlling for individual variables related to tooth loss.

## Methods

The study was designed as a cross-sectional multilevel study. In addition to individual variables, contextual variables were taken into account to explain an outcome assessed at the individual level. The individual variables were obtained from the Brazilian Oral Health Survey - SBBrasil 2010 [18], and the contextual variables were collected at the municipal level from official public databases.

SBBrasil 2010 represented a national epidemiological survey on oral health funded by the Ministry of Health. For representation of the complete Brazilian population, individuals aged 5 and 12 years and those in age groups 15–19, 35–44, and 65–74 years from 177 Brazilian municipalities were evaluated. Sampling was carried out at different domains of the state capitals, federal district, and municipalities within defined geopolitical macro-regions (North, Northeast, Central West, Southeast, and South), using probabilistic sampling in multiple stages with a Design Effect (DEFF) of 2. The primary sampling units were: (a) municipality, for the interior of the regions, and (b) census tract for the state capitals. Interviews and oral examinations were conducted in the subjects’ homes. Oral examinations were performed under natural light, by trained and calibrated examiners (Kappa ≥0.65), using a handheld computer to record the data. The diagnostic criteria of Oral Health Surveys: Basic methods (4th edition) from World Health Organization (WHO) were used [19]. In addition to assessment of the individual’s oral condition, interview was conducted with each household and comprised questions related to the socioeconomic profile of the family, use of dental services, self-reported oral morbidity, and self-perception of oral health. Details of the methodology used in SBBrasil 2010 have been described in a previous study [20]. In the present study, data of 9779 individuals in SBBrasil 2010 between the ages of 35 and 44 years were used, which is the standard age group for evaluation of oral health conditions in adults [19].

Contextual variables were collected from official public databases for each of 177 participating municipalities of SBBrasil 2010: Demographic census of 2010 by the Brazilian Institute of Geography and Statistics (IBGE) [21]; Atlas Brazil of the United Nations Development Program (UNDP) [22]; National Survey of Basic Sanitation of IBGE [23]; and the Department of Informatics of the Unified Health System (DATASUS) [24]. In the databases of Atlas Brazil [22] and National Survey of Basic Sanitation [23], data of IBGE 2010 demographic census were acquired between August 1, 2010 and October 30, 2010 from 316,574 census tracts with predefined territorial boundaries [21].

In this study, the dependent variable was total number of missing teeth defined as any natural tooth missing due to extraction, for any reason corresponding to codes 4 and 5 of the DMFT index (Decayed, Missing and Filled Teeth) [19]. This was assessed according to its discrete numerical nature whose values are whole numbers (counts).

**Fig. 1:**
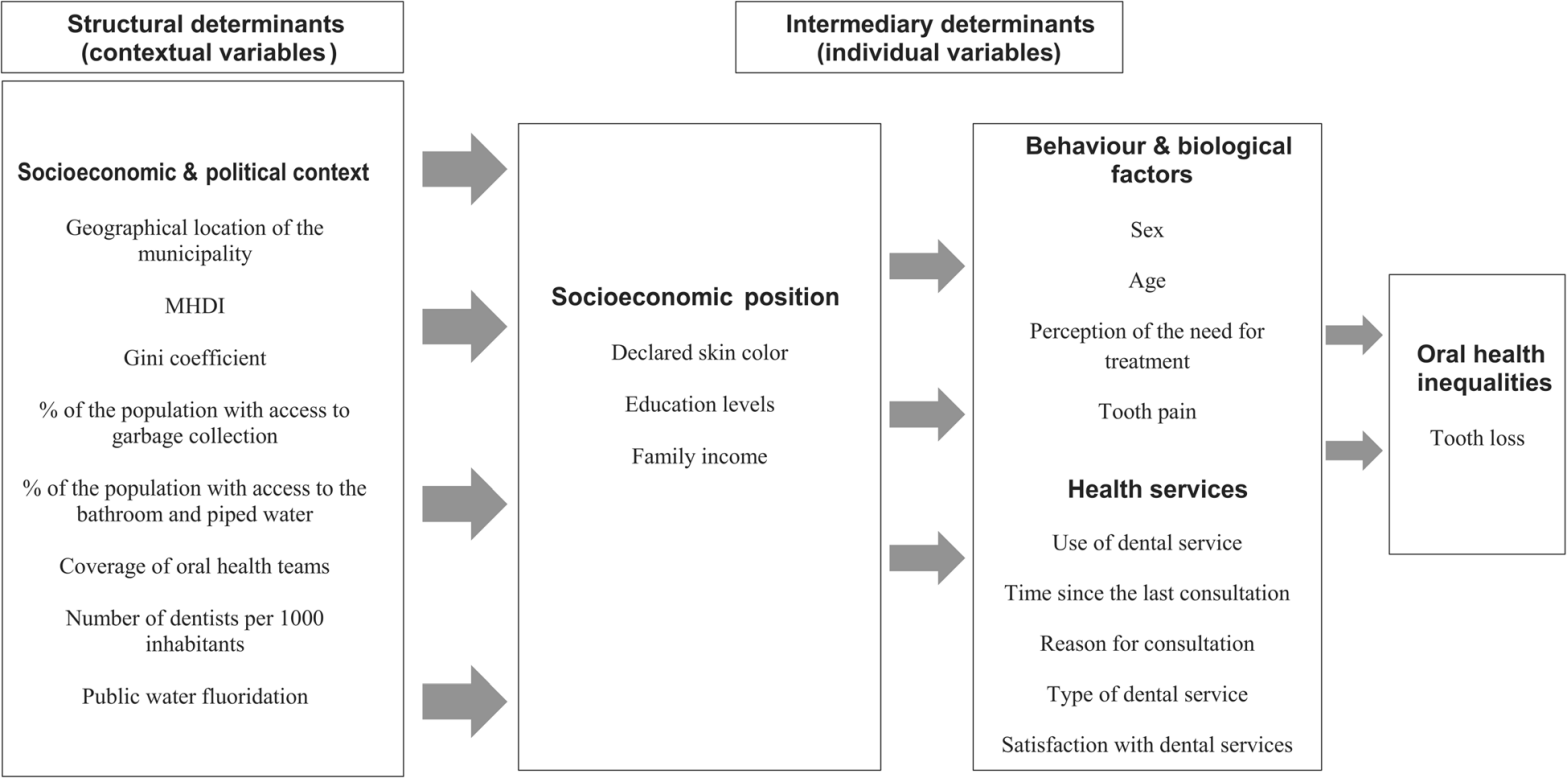
Conceptual model for the inequities of oral health adapted from Watt & Sheiham (2012)

The conceptual model for inequities in oral health of Watt & Sheiham (2012) [25] was building based on *Conceptual Framework for Action on the Social Determinants of Health* (CSDH) [26]. In our study, that model was used for the grouping of contextual and individual independent variables. This theoretical model takes into account the social determinants of inequalities in oral health, in contrast to preventive approaches, that focus on the behavioral changes of the individual. According to this conceptual model, the variables that influence the oral health can be grouped into structural determinants (socioeconomic & political context) and intermediary determinants (socioeconomic position, behavioral & biological factors, and health services) (Fig. 1).

In the socioeconomic & political context, all contextual variables were included: geographical location of the municipality (capital; interior) [18], Municipal Human Development Index (MHDI) (very high; high; medium/low) [22], Gini coefficient [22], percentage of the population with access to garbage collection [21], percentage of the population with access to a bathroom and piped water [21], coverage of oral health teams [24], number of dentists per 1000 inhabitants [24], and public water fluoridation (yes; no) [23]. MHDI reflects composite information on income, education level, and longevity in each municipality, and the scores are on a scale from 0 to 1, where higher values reflect a higher level of human development. Gini coefficient measures inequality in income distribution on a scale from 0 (absolute equality) to 1 (absolute inequality) [22]. The percentage of the population with access to garbage collection refers to the proportion of the population of each municipality with access to public garbage collection services [21]. The percentage of the population with access to a bathroom and piped water refers to the proportion of households in the municipality with simultaneous access to water supply (running water) by the distribution network, and bathroom or toilet facilities exclusively for residents [21]. The coverage of oral health teams refers to the proportion of the population in the municipalities that receive primary care of oral health teams [24]. The public water fluoridation classification used in this study was performed according to National Survey of Basic Sanitation from IBGE [23], which is exclusively based on information provided by sanitation companies. All contextual variables were analyzed as quantitative data expressed as numbers, except variables of the geographic location of the municipality, MHDI, and public water fluoridation.

In the socioeconomic position, individual variables were included as follows: declared skin color (white; yellow/black/brown/indigenous), education level (years of study), and family income in USD (> 2557; 853–2556; 285–852; ≤ 284); and the minimum wage at the time of data collection was USD 290.0.

In relation to behavioral & biological factors, individual variables were included as follows: sex (female; male), age (years), self-perception of the need for treatment (yes; no), and pain in the teeth and gums in the last 6 months (no; yes). Also at this level, considering health services, the following individual variables were included: previous use of dental service (yes; no), time since last consultation (≤ 1 year; > 1 year; no previous use of dental service), reason for consultation (review/prevention; oral health problems; no previous use of dental service), type of dental service (public; not public; no previous use of dental service), and satisfaction with dental services (satisfied; dissatisfied; no previous use of dental service).

### Analyses

To explore the dependent variable, a map was drawn with the average number of lost teeth for each one of the five Brazilian geopolitical macroregions, state capitals, and federal district. For each Brazilian macroregion, besides mean teeth lost, a confidence interval of 95% (95% CI) was estimate corrected by DEFF. Geographic Information System (GIS)-based Quantum GIS Software (QGIS; General Public License; GNU), which is freely available online, was used for manipulation of spatial data and construction of a map.

The data relating the individual and contextual variables was initially organized in the statistical software *Predictive Analytics Software* (SPSS/PASW®) version 18.0 for Windows®. The descriptive analyses of the contextual variables used only the municipal data. The descriptive analysis of the individual variables was conducted according to the need of correction for the effect of sample design, because they are from samples by conglomerates. For such, the *Complex Samples* module was used, considering the weights resulting from the sampling process adopted. Measures of central tendency and variability were estimated for the numerical independent variables and simple (n) and relative (%) frequencies for categorical independent variables. The association between the total number of lost teeth and the individual characteristics was verified by the non-parametric tests: Spearman correlation (ƿ) for numerical independent variables; Mann-Whitney test for dichotomous independent variables; and Kruskall-Wallis test for the polytomous independent variables.

The data was exported to the STATA® software, version 14.0, and the Multilevel Hierarchical Negative Binomial Regression (stepwise backward method) model was used with use of contextual and individual data. The Negative Binomial Regression model is appropriate when the dependent variable is quantitative and with non-negative, integer values (counting data) and when there is overdispersion in the data (the variance of the dependent variable is greater than the mean) [27]. Before starting the modeling, the adequacy of the dependent variable for this regression modality was verified and confirmed. For estimation of adjustment between outcome (total number of teeth lost) and the independent variables from first (contextual) and second (individual) levels of analysis, the fixed effect model was used [28]. Initially, an empty model was used with only a random intercept and the dependent variable, without the others variables. Subsequently, a hierarchical block design was used as proposed by the adopted theoretical model [25] (Fig. 1). Model 1 included only the contextual variables (socioeconomic & political context). All eight contextual variables adopted in our study were included in this first model. Adjustment was made in Model 1 and only the contextual variables that were significantly associated with the outcome (*p* ≤ 0.05) were maintained. From the second model, the individual variables were taken into account. Model 2 kept the contextual variables adjusted in model 1, and added the socioeconomic position. This model was also adjusted (*p* ≤ 0.05). Model 3 comprised the variables adjusted in models 1 and 2 and added behavioral & biological factors and health services. This final model was adjusted again (p ≤ 0.05). The *menbreg, irr* function was used to obtain the Mean Ratio (MR) and its 95% CI. Moreover, a sensitivity analysis was performed using a multilevel logistic regression model. In order to accomplish this, a dependent variable *number of missing teeth* was dichotomized by its median (under and above median points). A supplementary file exhibits findings from this analysis (Table S1).

The analysis of the fit of the models was performed through *Deviance*, obtained through the Log Likelihood multiplied by (− 2), where it is expected that there will be significant differences between the models (difference greater than 3.84) [29]. In addition, multicollinearity was tested by verifying the correlations of independent variables, with no values above 0.7 being identified. We also conducted a comparison between both Brazilian adults included and excluded of the final analysis due to losses of the independent variables. A supplementary file shows findings of these analyses (Table S2).

SBBrasil 2010 was conducted according to the ethical principles of the Resolution of the National Health Council (CNS; number 196/96), related to research on human beings; it was approved by the Research Ethics Committee of the Ministry of Health and registered at the National Research Ethics Committee of Brazil (CONEP) (CNS approval number: 15.498/2009). All participants of this study signed the written informed consent form [20].

## Results

**Fig. 2:**
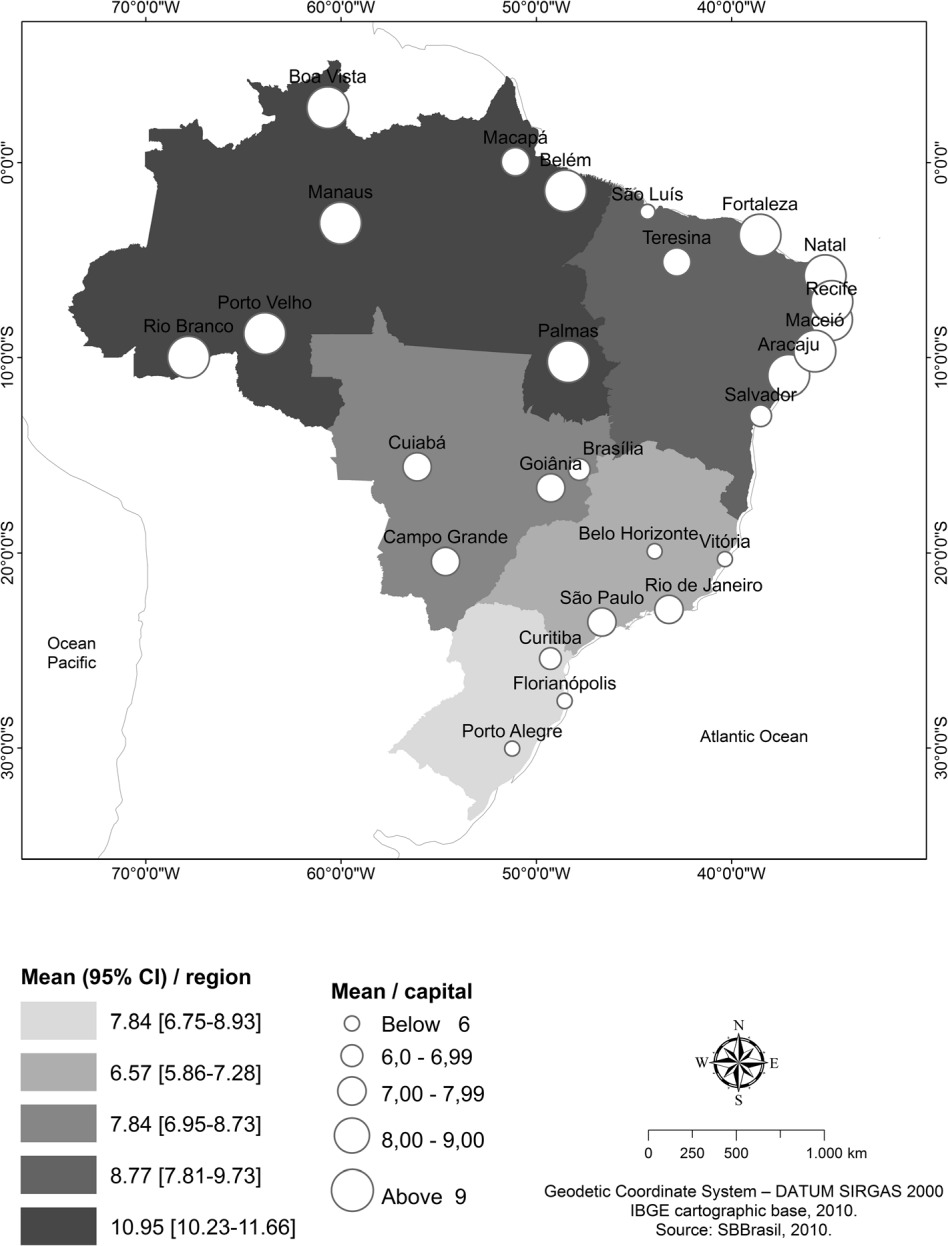
The mean number of missing teeth in the residents of Brazilian geopolitical macroregions (*n* = 9564). SBBrasil Project, 2010

Among the 9779 adult individuals of SBBrasil 2010, 215 (2.2%) were excluded due to no information of the dependent variable. Finally, a total of 9564 individuals were included in the study. The average number of adults evaluated in each Brazilian municipality was 54.03 (± 97.92), ranging from 3 to 488 individuals. The median tooth loss was 6.0, while the mean tooth loss was 7.57 (95% CI: 7.1–8.1) teeth, with higher values attained in the North (10.95) and Northeast (8.77) regions. Among the state capitals, tooth loss was lower in Vitória, Espírito Santo (4.23), followed in increasing order by Porto Alegre, Rio Grande do Sul (4.29), Belo Horizonte, Minas Gerais (5.03), and Florianópolis, Santa Catarina (5.13) (Fig. 2).

In what it refers to contextual variables, among the 177 municipalities analyzed, 85% were municipalities located in interior regions of the state, one third had no fluoridated water, and the average Gini coefficient was 0.62 (± 0.12). Mean tooth loss in adults from interior municipalities was 7.75, while adult city dwellers exhibited mean tooth loss of 6.45. In municipalities without fluoridated water, the mean number of missing teeth in adult population was 10.53, while for municipalities with fluoridated water, this mean was 7.53. The mean number of missing teeth was 5.62, 8.61, and 10.17 in adults from municipalities with very high, high, and medium/low MHDI, respectively.

In the descriptive analysis of the individual variables, there was a predominance of females and those who self-declared themselves as yellow/black/brown/indigenous. The mean age of adult individuals was 39.39 years (± 3.08), and the average years of education was 8.64 (± 3.93). Most of the adults utilized public dental services and self-perceived the need for dental treatment (Table 1). The bivariate analysis can also be observed in Table 1.

**Table 1:** Distribution of adults (*n* = 9564) according to the mean number of tooth loss. SBBrasil Project, 2010

Variables	n	%	Percentile			***p*** value
			25	50 (median)	75	
***Intermediary determinants***						
*Socioeconomic position*						
Declared skin color						
White	4049	47.6	1.0	4.0	10.0	< 0.001**
Yellow/Black/Brown/Indigenous	5515	52.4	3.0	7.0	13.0	
Education levels (in years)^a^*	$\overline{\boldsymbol{x}}$ (SD) = 8.64 (3.93)					< 0.001^#^
Family income (in US dollars)^b^						
> 2557	505	3.5	0.0	1.0	4.0	< 0.001†
853–2556	2741	29.3	1.0	4.0	9.0	
285–852	4687	53.4	3.0	7.0	13.0	
≤ 284	1404	13.8	4.0	8.0	16.0	
*Behavioral & biological factors*						
Sex						
Female	6287	62.3	2.0	6.0	12.0	< 0.001**
Male	3277	37.7	2.0	5.0	11.0	
Age (in years) ^a^	$\overline{\boldsymbol{x}}$ (SD) = 39.39 (3.08)					< 0.001^#^
Perception of the need for treatment^b^						
Yes	7360	77.0	2.0	6.0	12.0	< 0.001**
No	1999	23.0	1.0	4.0	12.0	
Tooth pain^b^						
No	7151	72.8	2.0	5.0	12.0	< 0.001**
Yes	2344	27.2	3.0	6.0	12.0	
*Health services*						
Previous use of dental service^b^						
Yes	8837	92.2	2.0	6.0	11.0	0.013**
No	672	7.8	2.0	6.0	16.0	
Time since the last consultation^b^						
≤ 1 year	4446	45.4	2.0	5.0	10.0	< 0.001†
> 1 year	4293	46.8	2.0	6.0	13.0	
No previous use of dental service	672	7.8	2.0	6.0	16.0	
Reason for consultation^b^						
Review/prevention	1910	19.4	0.0	3.0	7.0	< 0.001†
Oral health problems	6893	72.8	3.0	7.0	12.0	
No previous use of dental service	672	7.8	2.0	6.0	16.0	
Type of dental service^b^						
Public	5288	57.2	1.0	5.0	10.0	< 0.001†
Not public	3524	35.0	3.0	7.0	13.0	
No previous use of dental service	672	7.8	2.0	6.0	16.0	
Satisfaction with dental services^b^						
Satisfied	7373	78.4	2.0	6.0	11.0	< 0.001†
Dissatisfied	1404	13.8	3.0	6.0	12.0	
No previous use of dental service	672	7.8	2.0	6.0	16.0	

^a^Numerical variables

^b^Variation at *n* = 9,564. Due to loss of information

** *P* value calculated by the nonparametric Mann-Whitney test

^#^*P* value calculated by the Spearman correlation (ƿ)

† *P* value calculated by the nonparametric Kruskall-Wallis test

The results of multilevel hierarchical negative binomial regression analysis are shown in Table 2. With regard to contextual variables, tooth loss was higher in the municipalities with high or medium/low MHDI score (MR = 1.34 and 1.46 respectively). The average dental loss was increased 27% in the municipalities without fluoridated water, as compared to that in the municipalities with public water fluoridation (MR = 1.27). With regard to individual variables, the average number of tooth loss was higher in declared to be yellow / black / brown / indigenous (MR = 1.06), and lower among those with higher education levels (MR = 0.93). The average dental loss was higher in the individuals with family income of ≤ USD 2556.00 than in those with family income of ≥ USD 2557.00. In addition, it was lower in the male individuals than in the female individuals, which represents protection against tooth loss in male adults (MR = 0.87). The total number of missing teeth was higher among the older individuals (MR = 1.09) who never visited the dentist (MR = 1.42), those who received dental service more than 1 year ago (MR = 1.05), and those with last visit to the dentist due to oral health problems (MR = 1.42). Deviance was significantly reduced among the different models after adding each new block of variables.

**Table 2:** Multilevel hierarchical negative binomial regression of the tooth loss in adults (*n*= 9139). SBBrasil Project, 2010

***Variables***	Model 1		Model 2		Model 3	
	MR (95% CI)	***p*** value	MR (95% CI)	***p*** value	MR (95% CI)	***p*** value
***Structural determinants***						
*Socioeconomic & political context*						
MHDI						
Very high	Ref.		Ref.		Ref.	
High	1.46 (1.19–1.79)	< 0.001	1.35 (1.10–1.65)	0.004	1.34 (1.09–1.65)	0.006
Medium/low	1.80 (1.44–2.24)	< 0.001	1.46 (1.17–1.81)	0.001	1.46 (1.17–1.83)	0.001
Public water fluoridation						
Yes	Ref.	0.038	Ref.	0.001	Ref.	< 0.001
No	1.15 (1.01–1.32)		1.24 (1.09–1.41)		1.27 (1.11–1.45)	
***Intermediary determinants***						
*Socioeconomic position*						
Declared skin color						
White			Ref.		Ref.	
Yellow/Black/Brown/Indigenous			1.07 (1.02–1.12)	0.004	1.06 (1.02–1.11)	0.007
Education levels (in years)			0.92 (0.92–0.93)	< 0.001	0.93 (0.93–0.94)	< 0.001
Family income (in US dollars)						
> 2557			Ref.		Ref.	
853–2556			1.58 (1.42–1.75)	< 0.001	1.59 (1.44–1.76)	< 0.001
285–852			1.89 (1.71–2.10)	< 0.001	1.90 (1.72–2.10)	< 0.001
≤ 284			1.93 (1.72–2.16)	< 0.001	1.97 (1.76–2.20)	< 0.001
*Behavioral & biological factors*						
Sex						
Female					Ref.	
Male					0.84 (0.80–0.87)	< 0.001
Age (in years)					1.09 (1.09–1.10)	< 0.001
*Health services*						
Previous use of dental service						
Yes					Ref.	
No					1.42 (1.30–1.56)	< 0.001
Time since the last consultation						
≤ 1 year					Ref.	
> 1 year					1.05 (1.01–1.10)	0.012
No previous use of dental service					1.42 (1.30–1.56)	< 0.001
Reason for consultation						
Review/prevention					Ref.	
Oral health problems					1.42 (1.35–1.50)	< 0.001
No previous use of dental service					1.42 (1.30–1.56)	< 0.001

Empty model: *Deviance* = 59956.396

Model 1: *Deviance* = 59913.184

Model 2: *Deviance* =56976.56

Model 3: *Deviance* =55016.952

*MR* Mean ratio

*Ref.* Reference category

## Discussion

A higher tooth loss was observed among the residents of municipalities with high or medium/low MHDI and who did not have public water fluoridation, even after adjustment for individual variables. Among the individual variables, the number of lost teeth was influenced by declared skin color, education levels, income, sex, age, previous use of dental service, time elapsed since the last dental consultation and the reason for this consultation. In general, the highest number of missing teeth was related to unfavorable individual and contextual conditions.

The mean number of tooth loss among Brazilian adults was 7.57. Historically, a reduction in the number of lost teeth among Brazilian adults has been observed [18, 30, 31]. However, the number of lost teeth remains higher in less developed regions of the country (North and Northeast - 10.95 - 8.77). It should be emphasized that this pattern of regional differences has remained over time, in all age ranges [18, 30, 31]. In addition, levels of tooth loss among adults in Brazil are still higher than those observed in developed countries such as Canada (6.7) [32], Bulgaria (6.7) [33] and Ireland (5.7) [34]. This variation in the number of lost teeth for the different localities reinforces the idea of contextual influences on tooth loss and validates the findings of our study that unfavorable contexts increase the occurrence of tooth loss.

The contextual variables MHDI and public water fluoridation remained associated with the number of lost teeth, even after adjustment for important individual variables known to be associated to tooth loss. The association between contextual variables and the presence of functional dentition [14, 15], number of self-reported teeth [7, 16, 32, 35] and edentulism [13] has already been reported among adults. However, no previous studies have evaluated this relationship considering the number of lost teeth as numerical variable. This option offers the advantage of observing the magnitude of the impact per number of lost teeth, without the need for categorization of the variable, this allows the analysis to be more sensitive.

Adults living in municipalities with high or medium/low MHDI had a higher number of lost teeth compared to adults living in municipalities that had very high MHDI. Previous studies had already verified the effect of MHDI on the higher prevalence of functional dentition [14] and lower need for dental treatment among adults [36]. Municipalities that have greater MHDI possibly offer better opportunities for the maintenance of oral health, especially through increased access to dental services [35]. Furthermore, it is known that other important aspects for the maintenance of dentition, such as higher education levels, better eating habits, greater access to information and provision of health services, are commonly more available in developed areas [35, 37].

The public water fluoridation was associated with a lower number of lost teeth, which corroborates previous studies [14–16]. The effect of water fluoridation in reducing the prevalence and incidence of dental caries [38], the main cause of tooth loss [2], is widely recognized. Although we did not determine the time of availability of fluoride in the water supply to the municipalities, we believe individuals may benefit from access to fluoridated water throughout their life, rather than at a specific time point. The results emphasize the importance of water fluoridation as a public health measure. It was found that the impact of the addition of fluoride in the public water supply is higher for individuals of lower socioeconomic level [39], which reinforces the importance of this measure as a way to compensate for inequalities in oral health [38].

In relation to individual variables, adults with higher education levels and with higher income had lower tooth loss. This association is consistent in the national [2, 3, 11, 14–16] and international literature [5, 7, 32, 33]. Adults who declare being yellow, black, brown or indigenous displayed a higher tooth loss compared to whites. A study conducted among Brazilian adults also found racial inequity associated with tooth loss, with greater vulnerability of blacks and browns compared to whites [40]. The variable declared skin color was allocated in the block of socioeconomic position, since genetic studies have identified that there may not be a significant association between skin color and genomic ancestry [41, 42]. Thus, in countries where there is a large miscegenation, as is the case for Brazil, the variable of declared skin color seems to reflect more a socioeconomic condition than a genetic aspect.

A lower number of tooth loss among men [2, 3, 10, 11, 14–16, 33] and higher among older individuals [3, 5, 11, 14–16, 33] had already been observed in previous studies. Moreover, the number of missing teeth was higher among adults who never used dental services, who used dental services more than a year ago and whose reason for consultation was for oral health problems, to the detriment of preventive use. Previous studies have also reported an association between the use of dental services in the last year and lower tooth loss [5, 14, 15]. The adult population, especially workers, may have difficulties in accessing oral health services during the normal business hours [43]. This possibly leads these individuals to use dental services sporadically, seeking care for urgent dental conditions, in which restorative treatments may not be an option, increasing the likelihood of tooth loss. In addition, the preventive use (review/follow-up/routine/check-up) of dental services is considered an indicator of oral health, and the most conservative dental treatments were performed in patients who used them regularly [44]. A prospective cohort study carried out in New Zealand found that adults who routinely used dental services had higher levels of oral health, with less decayed and lost teeth [45]. Based on these facts, access to dental services on a regular basis should be encouraged in order to reduce the number of tooth loss among adults.

Our study has some limitations that should be highlighted. First, tooth loss was measured crosswise but it reflects the disease accumulation throughout life. In this way, we cannot establish a temporal relationship between tooth loss outcome and the others independent variables investigated in this study. Second, secondary data were analyzed and, consequently, important tooth loss risk factors, such as occurrence of systemic chronic conditions and lifestyle factors were not assessed once SBBrasil (2010) did not measure those characteristics. Longitudinal studies are needed to better clarify this issue. In the other hand, the present study included a representative sample of Brazilian adult population. Moreover, the multilevel approach of the variables allowed identifies the contribution of each block of variables to tooth loss. With this done, it was realized the influence of both individual and contextual factors on tooth loss outcome. Such effects were confirmed by using sensitivity analysis, which even changing the classification of the dependent variable and the type of analysis, it was still found the same contextual variables associated with tooth loss parameter. The random effect to contextual variables and for the individual variable sex was tested. However, there was no note any significant improvement in model adjustment.

## Conclusion

The contextual variables MHDI and public water fluoridation remained associated with the number of lost teeth among adults, even after controlling for important individual variables known to be associated with tooth loss. Generally, a higher number of missing teeth was associated with unfavorable contextual and individual factors: lower MHDI, absence of the public water fluoridation, less education levels, low family income, no previous use of dental service and greater time elapsed since the last dental consultation. Thus, the findings of this study reinforce the impact of social inequality in tooth loss and require a reflection on the need for expansion and better organization of dental services in order to promote equity between individuals.

## Supplementary information

**Additional file 1: Table S1.** Sensitivity analysis performed with multilevel logistic regression to tooth loss (dichotomized by median) in Brazilian adults (*n* = 9139). SBBrasil Project, 2010. **Table S2.** Analysis of the differential loss among included and excluded Brazilian adults of the final regression model due to missing of independent variables. SBBrasil Project, 2010.

## Data Availability

The datasets used and/or analyzed during the current study are available from the corresponding author on reasonable request.

## Abbreviations

CNS: National Health Council
CONEP: National Research Ethics Committee of Brazil
CSDH: Conceptual Framework for Action on the Social Determinants of Health
DATASUS: Department of Informatics of the Unified Health System
DEFF: Design Effect
DMFT: Decayed, Missing and Filled Teeth
GIS: Geographic Information System
IBGE: Brazilian Institute of Geography and Statistics
MHDI: Municipal Human Development Index
MR: Mean Ratio
QGIS: Quantum GIS Software
SBBrasil 2010: Brazilian Oral Health Survey
SPSS/PASW®: Predictive Analytics Software
UNDP: Atlas Brazil of the United Nations Development Program
WHO: World Health Organization
