# Supplementary material for: Contextual and individual determinants of tooth loss in adults: a multilevel study
Source: BMC Oral Health. 2020 Mar 17;20:73. doi: 10.1186/s12903-020-1057-1 (PMC7076961; doi:10.1186/s12903-020-1057-1)
Supplement: Supplementary file 1 — Additional file 1: Table S1. Sensitivity analysis performed with multilevel logistic regression to tooth loss (dichotomized by median) in Brazilian adults (n = 9139). SBBrasil Project, 2010. Table S2. Analysis of the differential loss among included and excluded Brazilian adults of the final regression model due to missing of independent variables. SBBrasil Project, 2010. [file 12903_2020_1057_MOESM1_ESM.docx]

**Supplementary material**

**Table S1**. Sensitivity analysis performed with multilevel logistic regression to tooth loss (dichotomized by median) in Brazilian adults (n = 9,139). SBBrasil Project, 2010.

| ***VARIABLES*** | **OR (95% CI)** | **p value** |
| --- | --- | --- |
| ***STRUCTURAL DETERMINANTS*** | |  |
| *Socioeconomic & political context* | |  |
| MHDI |  |  |
| Very high | Ref. |  |
| High | 1.89 (1.21-2.95) | 0.005 |
| Medium/low | 2.28 (1.41-3.70) | 0.001 |
| Public water fluoridation |  |  |
| Yes | Ref. |  |
| No | 1.71 (1.27-2.29) | < 0.001 |
| ***INTERMEDIARY DETERMINANTS*** | |  |
| *Socioeconomic position* |  |  |
| Declared skin color |  |  |
| White | Ref. |  |
| Yellow/Black/Brown/Indigenous | 1.17 (1.06-1.30) | 0.003 |
| Education levels (in years) | 0.88 (0.87-0.89) | < 0.001 |
| Family income (in US dollars) |  |  |
| > 2557 | Ref. |  |
| 853-2556 | 2.02 (1.53-2.69) | < 0.001 |
| 285-852 | 3.04 (2.29-4.04) | < 0.001 |
| ≤ 284 | 3.40 (2.50-4.63) | < 0.001 |
| *Behavioral & biological factors* | |  |
| Sex |  |  |
| Female | Ref. |  |
| Male | 0.67 (0.61-0.74) | < 0.001 |
| Age (in years) | 1.19 (1.17-1.21) | < 0.001 |
| *Health services* |  |  |
| Previous use of dental service |  |  |
| Yes | Ref. |  |
| No | 1.32 (1.05-1.66) | 0.015 |
| Time since the last consultation |  |  |
| ≤ 1 year | Ref. |  |
| > 1 year | 0.97 (0.88-1.07) | 0.589 |
| No previous use of dental service | 1.32 (1.05-1.66) | 0.015 |
| Reason for consultation |  |  |
| Review/prevention | Ref. |  |
| Oral health problems | 1.91 (1.68-2.18) | < 0.001 |
| No previous use of dental service | 1.32 (1.05-1.66) | 0.015 |

OR = Odss Ratio

Ref. = Reference category

**Table S2:** Analysis of the differential loss among included and excluded Brazilian adults of the final regression model due to missing of independent variables. SBBrasil Project, 2010.

| ***VARIABLES*** | | **Included**  (n=9,139) | **Excluded**  (n=425) | **p value** |
| --- | --- | --- | --- | --- |
|  | | % | % |  |
| Declared skin color | |  |  |  |
| White | | 42.5 | 39.7 | 0.057^§^ |
| Sex | |  |  |  |
| Female | | 65.9 | 62.6 | 0.160^§^ |
| Perception of the need for treatment | |  |  |  |
| Yes | | 78.7 | 78.1 | 0.800^§^ |
| Tooth pain | |  |  |  |
| Yes | | 24.7 | 25.0 | 0.088^§^ |
| Previous use of dental service | |  |  |  |
| Yes | | 92.9 | 93.2 | 0.812^§^ |
| Time since the last consultation | |  |  |  |
| ≤ 1 year | | 47.4 | 42.3 | 0.160^§^ |
| Reason for consultation | |  |  |  |
| Review/prevention | | 72.6 | 76.5 | 0.160^§^ |
| Type of dental service | |  |  |  |
| Public | | 55.7 | 57.1 | 0.800^§^ |
| Satisfaction with dental services | |  |  |  |
| Satisfied | | 78.0 | 78.9 | 0.800^§^ |
| Education levels (in years) | |  |  |  |
|  | 25 | 6.0 | 5.0 | < 0.001* |
| Percentile | 50 (median) | 9.0 | 8.0 |  |
|  | 75 | 11.0 | 11.0 |  |
| Age (in years) | |  |  |  |
|  | 25 | 37.0 | 37.0 | 0.005* |
| Percentile | 50 (median) | 39.0 | 40.0 |  |
|  | 75 | 42.0 | 43.0 |  |
| Number of missing teeth | |  |  |  |
|  | 25 | 2.0 | 2.0 | 0.108* |
| Percentile | 50 (median) | 6.0 | 6.0 |  |
|  | 75 | 12.0 | 13.0 |  |

^§^ P value calculated by the Pearson chi-square test.

* P value calculated by the nonparametric Mann-Whitney test.
